# Ion correlation and negative lithium transference in polyelectrolyte solutions†

**DOI:** 10.1039/d3sc01224g

**Published:** 2023-05-16

**Authors:** Helen K. Bergstrom, Kara D. Fong, David M. Halat, Carl A. Karouta, Hasan C. Celik, Jeffrey A. Reimer, Bryan D. McCloskey

**Affiliations:** a Department of Chemical & Biomolecular Engineering, University of California Berkeley CA 94720 USA helen_bergstrom@berkeley.edu bmcclosk@berkeley.edu; b Energy Storage and Distributed Resources Division, Lawrence Berkeley National Laboratory Berkeley CA 94720 USA; c Materials Sciences Division, Joint Center for Energy Storage Research, Lawrence Berkeley National Laboratory Berkeley CA 94720 USA; d College of Chemistry NMR Facility, University of California Berkeley CA 94720 USA

## Abstract

Polyelectrolyte solutions (PESs) recently have been proposed as high conductivity, high lithium transference number (*t*_+_) electrolytes where the majority of the ionic current is carried by the electrochemically active Li-ion. While PESs are intuitively appealing because anchoring the anion to a polymer backbone selectively slows down anionic motion and therefore increases *t*_+_, increasing the anion charge will act as a competing effect, decreasing *t*_+_. In this work we directly measure ion mobilities in a model non-aqueous polyelectrolyte solution using electrophoretic Nuclear Magnetic Resonance Spectroscopy (eNMR) to probe these competing effects. While previous studies that rely on ideal assumptions predict that PESs will have higher *t*_+_ than monomeric solutions, we demonstrate that below the entanglement limit, both conductivity and *t*_+_ decrease with increasing degree of polymerization. For polyanions of 10 or more repeat units, at 0.5 m Li^+^ we directly observe Li^+^ move in the “wrong direction” in an electric field, evidence of a negative transference number due to correlated motion through ion clustering. This is the first experimental observation of negative transference in a non-aqueous polyelectrolyte solution. We also demonstrate that *t*_+_ increases with increasing Li^+^ concentration. Using Onsager transport coefficients calculated from experimental data, and insights from previously published molecular dynamics studies we demonstrate that despite selectively slowing anion motion using polyanions, distinct anion–anion correlation through the polymer backbone and cation–anion correlation through ion aggregates reduce the *t*_+_ in non-entangled PESs. This leads us to conclude that short-chained polyelectrolyte solutions are not viable high transference number electrolytes. These results emphasize the importance of understanding the effects of ion-correlations when designing new concentrated electrolytes for improved battery performance.

## Abstract 2

We demonstrate that contrary to previous reports, transference number decreases with increasing degree of polymerization in non-aqueous lithium-bearing polyelectrolyte solutions that have been proposed as next-generation battery electrolytes.

## Graphical abstract

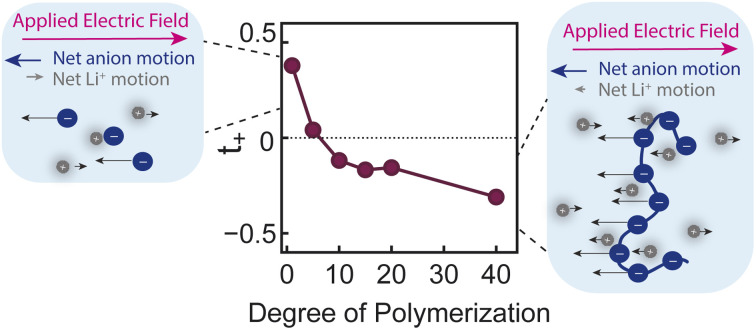

## 1 Introduction

While lithium-ion batteries (LIBs) have made significant market penetration into consumer electronics, distributed energy storage and electric vehicles, research has focused on increasing LIB energy density and attainable charging rates while mitigating safety risks to ensure continued adoption. In particular, in attempts to solve these problems, much effort has been devoted to engineering new classes of high performance electrolytes.^1^ Current state-of-the-art Li-ion batteries make use of dissociated binary lithium salts in a blend of liquid carbonate solvents. These liquid electrolyte systems suffer from low transference number (*t*_+_), meaning the majority of ionic conductivity results from motion of the anion rather than the electrochemically active Li^+^ ion. The resulting ionic concentration gradients in the electrolyte extend into the porous electrode and generate potential losses that limit the rate and efficiency of charging, limit material utilization, and increase the risk of short-circuiting through Li plating and dendrite growth.^1–3^ High Li^+^ transference number electrolytes (HTNEs), such as ceramic single ion conductors, swollen ionomers, and dry solid polyion electrolytes, carry the majority of current *via* mobile, coordinated Li^+^ species and are a promising strategy to reduce detrimental concentration gradients. However, current HTNEs are incompatible with conventional battery cell designs and manufacturing procedures, and their increased transference numbers typically come at the cost of substantially reduced ionic conductivity.^3^

Non-aqueous polyelectrolyte solutions (PESs) have been suggested as promising route to high *t*_+_, high conductivity electrolytes.^3–10^ Increasing *t*_+_ requires decreasing the mobility of the anion, which is dictated by both the diffusion of the anion as well as its net charge. PESs are intuitively appealing because anchoring the anion to a polymer backbone slows down the motion of the electrochemically inactive anion while maintaining higher ion conductivity through improved ion dissociation and solvent-mediated Li^+^ transport. In polyelectrolyte systems, however, increasing molecular weight both decreases polymer diffusion and increases charge, which will act as competing effects for *t*_+_. Experimental studies of PESs to date have focused on self-diffusion coefficients measurements or Bruce-Vincent type measurements to characterize transport and used ideal solution assumptions to claim high-transference numbers. Buss *et al.* reported ideal *t*_+_ values of 0.8 to 0.95 for a poly(allyl glycidyl ether-lithium sulfonate) in dimethyl sulfoxide. Dewing *et al.* reported ideal *t*_+_ values between 0.77 and 0.98 for poly(lithium bis-(nonenylmalonato)borate) in propylene carbonate and ionic conductivities of 0.3–0.47 mS cm^−1^. Both studies found increasing *t*_+_ with increasing polyanion molecular weight. While these reports are impressive, the ideal solution assumptions involved in these studies significantly limit the applicability of these results to understanding true transport properties in PESs. Recent molecular dynamic simulations of PESs have highlighted the critical importance of correlated ion motion in these systems and have called into question oligomeric PESs as a feasible strategy to achieving high *t*_+_ and conductivity electrolytes.^11,12^

Experimentally measuring rigorous transport coefficients, especially *t*_+_, is particularly challenging in PESs where synthetic scale limitations make traditional methods (*e.g.* the Hittorf method) unfeasible. In this work we seek to study the true non-ideal transport properties systematically as a function of polyanion molecular weight for a model battery-relevant polyelectrolyte solution system of lithium triflimide appended polystyrene dissolved in a liquid carbonate blend. Using electrophoretic Nuclear Magnetic Resonance Spectroscopy (eNMR) and electrochemical experiments, we rigorously characterize the transport properties, including the electrophoretic ion mobilities, conductivity, salt and self-diffusion coefficients, and *t*_+_ of these model PESs. We begin with a discussion of the synthesis and characterization of our model polyelectrolyte system in the oligomeric regime ranging from 1 to 40 repeat units. This molecular weight range was chosen to avoid the effects of entanglement and because we can achieve battery-relevant conductivities on the order of 1 mS cm^−1^ for oligomeric polyelectrolytes solutions. Next, we present measurements of the transport properties of electrolyte solutions made from these oligomers as a function of molecular weight and concentration. We demonstrate that despite selectively slowing anion motion through their incorporation in polyanion chains, anion–anion correlation through the polymer backbone and cation–anion correlation through ion aggregates reduce the *t*_+_ in non-entangled PESs. These findings confirm previous coarse-grained molecular dynamic models that highlighted the importance of these non-ideal ion correlations.^11,12^ This work represents the first rigorous characterization of transport properties for a battery-relevant polyelectrolyte solution and demonstrates that oligomeric PESs are not feasible high-transference number electrolytes. The novel eNMR methods and insights from the Onsager transport framework described herein have broad applicability to the design and study of liquid and polymer electrolytes.

## 2 Experimental materials & methods

### 2.1 Materials

Battery grade ethyl methyl carbonate (EMC) and ethylene carbonate (EC) were purchased from Gotion Inc. and directly transferred under inert atmosphere to an argon glovebox (Vacuum Atmospheres) kept below 5 ppm water and oxygen. Anhydrous dimethyl formamide (DMF) packed under inert atmosphere was purchased from Sigma and directly transferred into the glovebox. Lithium sulfonyl(trifluoromethane sulfonyl)imide styrene (STFSILi) was purchased from Specific Polymers and stored under argon atmosphere at 4 °C in an air-tight desiccator. Azobisisobutyronitrile (AIBN), 2-cyano-2-propyl benzodithioate (97%), and 2-(dodecylthiocarbonothioylthio)propionic acid (97%) were purchased from Sigma Aldrich. Blocbuilder MA was provided by Arkema. All chemicals were used as received without further purification. Lithium metal foil (0.75 mm thick) was obtained from MTI Corporation and lithium metal wire (3.2 mm diameter) was obtained from Alfa-Aeser. All lithium was brushed with nylon bristles to remove the native surface layer prior to use.

### 2.2 Oligomer synthesis

**Fig. 1:**
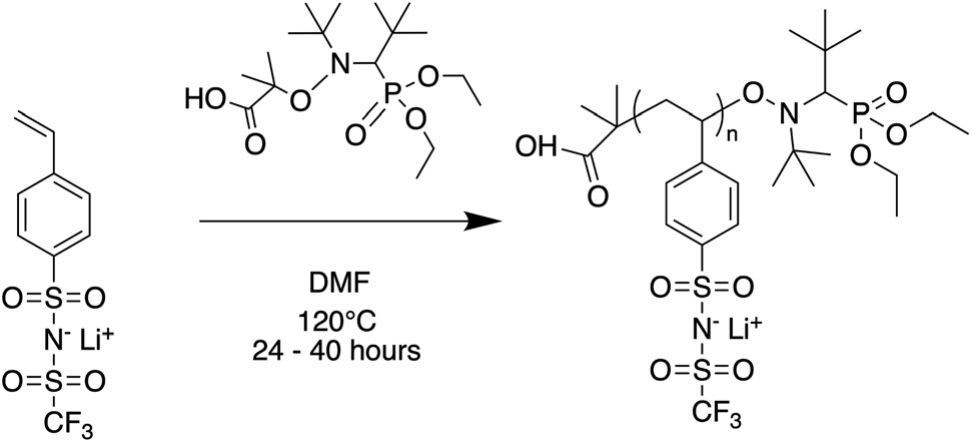
Nitroxide mediated polymerization of STFSI-Li.

Oligomers ranging from 5 to 40 repeat units were synthesized *via* nitroxide-mediated controlled radical polymerization (NMP) (Fig. 1). The authors note that these oligomers were also successfully synthesized using reversible addition fragmentation chain-transfer polymerization (RAFT), however yields were poor at the lowest degrees of polymerization (DP) and end-groups were difficult to fully remove (see ESI†). The molecular weight was varied by setting the molar ratio of STFSILi monomer to Blocbuilder MA equal to the desired DP. 5 grams (15.6 mmol) of STFSILi monomer and the appropriate amount of Blocbuilder MA for the desired DP were loaded into a round bottom flask inside an argon filled glovebox, dissolved in 9 mL of anhydrous DMF, and capped with a rubber septa before removal from the glovebox. All oligomerization reactions were carried out at 120 °C in an oil bath for 24–40 hours until quantitative conversion was reached as determined by NMR. The reaction mixture was precipitated and washed 3 times in diethyl ether. Due to sparing oligomer solubility in ether, the 5 and 10 repeat unit oligomers were directly dialysed in water using a 0.5–1 kDa cutoff membrane (Repligen Biotech CE) without precipitation. All oligomers were dissolved in water and lyophilized to obtain a fluffy yellow-beige powder. Prior to use in electrolytes, oligomers were dried at 75 °C in a vacuum oven over phosphorous pentoxide for 24 hours to remove trace water. Gel permeation chromatography (GPC) was used to determine molecular weight distributions and polydispersities on a Agilent 1260 Infinity Series GPC with Waters Styragel HR3 and HR4 columns and *N*-methyl-pyrrolidone with 0.05 M lithium bromide as the mobile phase. The GPC was calibrated with polystyrene standards.

### 2.3 Polyelectrolyte solution preparation & characterization

Polyelectrolyte solutions were prepared inside the glovebox using a moles of Li^+^ per kg solvent basis. All electrolyte solutions were made using a 3 : 7 EC : EMC ratio by weight. Solution densities were measured in an Anton Paar DMA 4101 oscillating U-tube density meter at 30 °C inside an inert glovebox. Each density measurement was performed in triplicate. Viscosity measurements were performed in triplicate in an electromagnetically spinning viscometer (EMS-1000s, Kyoto Electronics) spinning at 1000 rotations per minute at 30 °C. Samples were sealed inside air-tight vials inside the glovebox before transfer to the viscometer to ensure no moisture or air contamination.

### 2.4 Conductivity

Solution conductivity (*κ*) was measured using a Mettler Toledo InLab 751-4mm conductivity probe with blocking platinum electrodes inside the glovebox. The conductivity probe was calibrated using 84 μS cm^−1^, 1413 μS cm^−1^, and 12.88 mS cm^−1^ aqueous standards (Mettler Toledo) prior to bringing it inside the glovebox. Samples were maintained at 30 °C using a dry block (Torrey Pines). Solution temperatures were verified using a temperature sensor inside the cell and were always within ±0.5 °C of the set point. A 5% error is estimated for probe measurements based on replicate measurements and calibration error. Solution conductivity was also verified inside our fused electrophoretic NMR cells (P&L Scientific) using AC impedance spectroscopy (Bio-Logic SP-300 potentiostat) in the frequency range of 1 MHz to 100 mHz with a 10 mV AC amplitude. The cell constant of each NMR cell was measured using 84 μS cm^−1^, 1413 μS cm^−1^, and 12.88 mS cm^−1^ aqueous standards. Impedance data was tested for linearity using a Kramers–Kronig analysis and fit to a RQ equivalent circuit using the open-source Py-EIS package for Python.^13^

### 2.5 Concentration cells

Concentration cells were constructed inside a custom fabricated low-volume glass U-cell with a P4 glass frit (Adams & Chittenden).^14^ Concentration cell measurements were performed inside the argon glovebox with cell temperature maintained using a dry block with each U-cell equilibrated at 30 °C prior to electrolyte addition. Concentration cell measurements were constructed for 10 logarithmically spaced concentration combinations ranging from 0.05 m to 1.2 m Li^+^. For each measurement, one side of the U-cell contains 0.1 m Li^+^ polyelectrolyte in EC : EMC solution as a reference electrolyte while the other side contains electrolyte of varying concentration. U-cells were constructed using 750 μL of electrolyte added on each side of the glass frit before brushed lithium metal wire electrodes were immersed in the solution on each side. The open-circuit potential, *U*(*t*), was recorded over the course of 2 hours using a Bio-Logic VMP3-potentiostat with a 0.5 second sampling rate.

The change in liquid junction potential (*U*) across a concentration cell as a function of concentration is related to the transference number and thermodynamic factor 
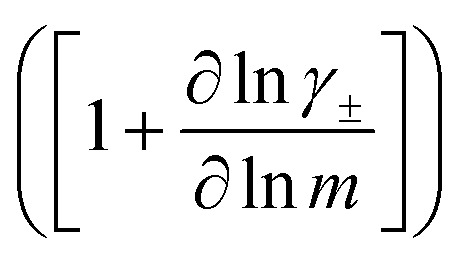
 according to1
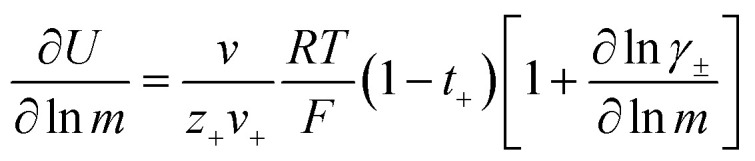
where *m* is the solution molality, *v*_i_ is the stoichiometric coefficient of species i, and *γ*_±_ is the molal activity coefficient.^15,16^

### 2.6 Restricted diffusion measurements

Restricted diffusion measurements were performed inside lithium symmetric coin cells. Ten layers of 19 mm disks of Celgard 2500 impregnated with electrolyte were sandwiched between 15 mm brushed lithium electrodes inside a CR2032 coin cell (Hohsen Corporation). To ensure good wetting of the electrode and separator, 5 μL of electrolyte was added in between each layer for a total added electrolyte volume of 55 μL. Each electrode stack was finished with a 0.5 mm thick stainless steel spacer and wave spring before crimping. Three replicate cells were made for each concentration. Cells were run inside an environmental chamber (Thermotron Inc.) maintained at 30 °C and allowed to equilibrate at open circuit potential for 2 hours prior to testing. The cell is then polarized at 50 mV for twelve hours to allow concentration gradients to build before allowing the cell to relax at open circuit for two hours with potential recorded every 0.5 seconds. The effective total salt diffusion coefficient (*D*_±_^eff^) can be obtained by fitting the voltage relaxation to eqn (2) where *l* is the electrode separation distance.^16–18^ The electrolyte total diffusion coefficient (*D*_±_) is obtained by simply multiplying *D*_±_^eff^ by the separator tortuosity. For the purposes of this work we use a tortuosity value for Celgard 2500 of 2.5 as measured by Landesfeind *et al.*^19^2
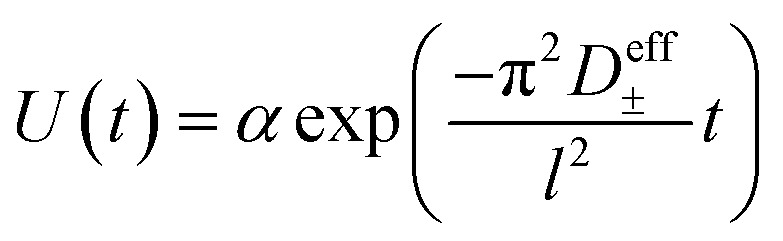


Our previous work emphasized the importance of the fitting window on the obtained *D*_±_, in particular that fits to times significantly greater that 4 times the characteristic diffusion time leads to unrealistically small diffusion coefficients due to significant interfacial instability-driven noise.^16^ To minimize the influence of interfacial noise at long times (low voltages), we logarithmically downsampled the data prior to fitting to the exponential in eqn (2).

### 2.7 Pulsed field gradient NMR

Self-diffusion coefficients of each species were measured using pulsed field gradient (PFG) NMR at a field strength of 9.4 T on a Bruker NEO 400 MHz spectrometer fitted with a 5 mm water-cooled double resonance broadband diffusion (diffBB) probe equipped with *z*-axis gradient capabilities up to 17 T m^−1^ and a variable temperature unit that was maintained at 30 °C throughout measurements. Temperature of the probe was calibrated using a 80% ethylene glycol – 20% deuterated dimethyl sulfoxide standard. The gradient was calibrated to known diffusion coefficients for ethylene glycol^20^ and 0.25 M LiCl in H_2_O.^21^ Samples were prepared inside the glovebox and sealed with an air-tight Teflon cap. The 90° pulse time and *T*1 relaxation time for each sample were measured and a repetition time of 5 seconds used for all experiments. A double stimulated echo bipolar gradient pulse sequence (Bruker pulse sequence diffDSTEAV3) with sin-bell magnetic field gradient pulses (SIN.100) was used in order to eliminate convection-based artifacts for all 3 measured nuclei (1H, 19F, 7Li).^22^ Eight dummy gradient pulses were applied at the beginning of each program prior to spectral acquisition to warm up the gradient amps. For each nucleus 16 linearly spaced gradient steps were acquired with the gradient parameters optimized such that the signal attenuates over at least one order of magnitude. Representative values for gradient and pulse program parameters are reported in the ESI.† PFG data was fit to the Stejskal–Tanner equation3
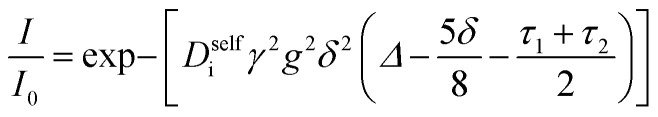
where *D*_i_^self^ is the self diffusion coefficient of species i, *γ* is the gyromagnetic ratio, *g* is the gradient strength including a correction for the sin-bell shape factor, *δ* is the gradient pulse duration, *Δ* is the drift delay, and *τ*_1_ and *τ*_2_ are the gradient recovery delays.^23^

### 2.8 Electrophoretic NMR

eNMR experiments were performed on the Bruker NEO 400 MHz magnet equipped with the diffBB probe described above with variable temperature control maintained at 30 °C throughout experiments. Temperature of the probe was calibrated as described above, and checked prior to measurements with a solution of pure ethylene glycol inside an eNMR cell. Electric field pulses were applied with a P&L eNMR 1000 electrophoretic high-voltage amplifier unit operating in voltage-controlled mode (P&L Scientific Instrument Services) based on the setup described in ref. 24 and ref. 25. eNMR amplifier pulses were controlled by incoming trigger pulses from the Bruker spectrometers to synchronize the electric field pulses with radio frequency (rf) and magnetic field gradient of the eNMR pulse program. Noise from rf pulses was suppressed using a two-stage electronic filter assembly – the first grounded on the NMR preamplifier and the second embedded in the eNMR cell holder provided by P&L. eNMR sample cells consisting of a 5 mm glass tube with palladium electrodes with an approximate inter-electrode distance of 3.35 cm (ref. 25) were purchased from P&L. Exact inter-electrode distances were calibrated by measuring the mobility of a 10 mM tetramethylammonium bromide solution in deuterated water and comparing to literature values.^24,25^ Calibrated cells were filled with ∼600 μL of electrolyte inside a glovebox and sealed with an air-tight Teflon plug. A convection-compensated double stimulated echo eNMR pulse sequence^26,27^ was used with bipolar electric field pulses lasting 50 ms each^28,29^ to reduce error induced by possible convection, electro-osmotic flow, and bubble formation. Representative values for gradient and pulse parameter values for each nucleus as well as a discussion of artifact reduction can be found in the ESI.† eNMR measurements were performed with voltage-controlled electric field pulses with the applied voltage range chosen on a per-sample basis. The lower end of the voltage range was selected such that a phase shift was discernible, ∼1°, while the upper voltage range was selected as the highest voltage before signal attenuation due to convection was observed.

eNMR experiments are non-equilibrium measurements of ion drift velocities in an electric field which manifests as a phase angle shift in the NMR signal. The phase shift (*Φ* − *Φ*_0_) is directly related to the drift velocity, *v* and magnetic field gradient parameters according to eqn (4).

4 (*Φ* − *Φ*_0_) = *γδΔgv*

The electrophoretic mobility of a species i (*μ*_i_) can then be related to the drift velocity at a given the electric field (*E*) according to eqn (5).5
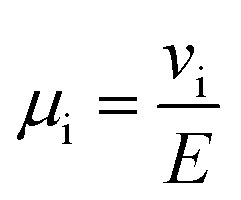


Determining appropriate phase angle of each spectra is essential for extracting mobility, however doing so systematically and without bias can be difficult.^30^ Schmidt *et al.* introduced a systematic method for obtaining phase angles using phase-sensitive spectral deconvolution assuming Lorentzian line shapes that is integrated into *eNMRpy*, an open-source python package for eNMR analysis.^30,31^ This method allows constraining the fit model such that peaks assigned to the same molecule have the same phase shift.^30^ In this work, we modified the *eNMRpy* source code to directly analyze files output from the Bruker NEO 400, extract relevant pulse program parameters and obtain phase angles for each spectra using Lorentzian fitting. For ^7^Li and ^19^F only one broad peak is observed and therefore no constraints are needed. However for ^1^H spectra, we apply constraints to the fitting such that the fitted phases are the same for all peaks belonging to EMC and to EC, respectively. We observed that line broadening had a noticeable effect on the phase fit. In order to standardize fit, we use a matched filter condition – such that line broadening was chosen to match the natural linewidth of the peak of interest. In order to ensure that our measurements were free of major artifacts, we compared conductivity of our solution obtained by impedance spectroscopy on each sample (see description above) to those calculated using our measured ion mobilities. The conductivity of the solution can be related to ion mobilities according to

6 *κ*^eNMR^ = *F*(*z*_+_*c*_+_*μ*_+_ + *z*_−_*c*_−_*μ*_−_)

Velocities from eNMR are all with reference to the stationary NMR probe. We can switch to a solvent velocity reference frame denoted by a superscript ‘0’ by subtracting the solvent velocity from other species velocities. In the case of the mixed-solvent system presented here, the mean solvent velocity is taken a mass-weighted average of the individual solvent velocities. In the case of zero solvent motion, the stationary and solvent reference frames will yield the same velocities and therefore the same transport properties. Finally to convert to a center of mass reference frame denoted by a superscript ‘COM’, we can calculate the center of mass velocity according to

7 *v*^COM^ = *ω*_+_*v*_+_ + *ω*_−_*v*_−_ + *ω*_0_*v*_0_

where *ω*_i_ is the mass fraction of species i in the solution.^16^

## 3 Results and discussion

### 3.1 Polyelectrolyte solution physical properties

We achieved varying degrees of polymerization between 5 and 40 repeat units of the STFSI-Li monomer as targeted. We selected this oligomeric range to reduce entanglement effects^32–34^ and to allow comparison with existing molecular dynamic simulations.^11^ GPC confirmed that we achieved unimodal size distributions with low polydispersity for all oligomers ranging from 5 to 40 repeat units in targeted length (see Table 1).

**Table 1:** Synthesized oligomer properties

Repeat units	Target molecular weight (g mol^−1^)	Polydispersity
1	321.22	—
5	1990	1.11
10	3596	1.11
15	5202	1.12
20	6808	1.14
40	13 233	1.22

**Fig. 2:**
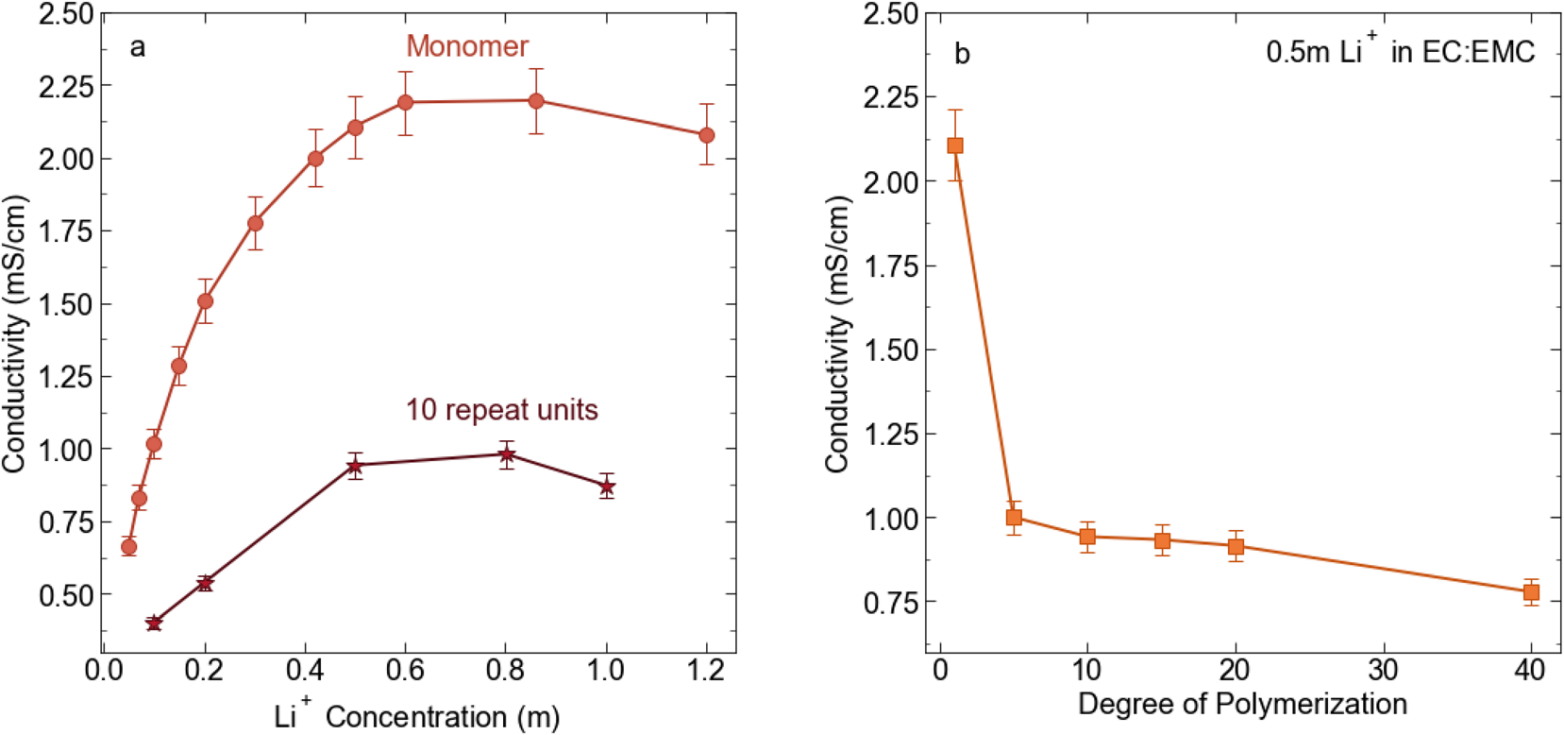
(a) Conductivity (mS cm^−1^) for STFSI-Li monomer in 3 : 7 EC : EMC at 30 °C *vs.* salt concentration (b) conductivity (mS cm^−1^) for 0.5 m Li^+^ in 3 : 7 EC : EMC at 30 °C as a function of poly-STFSI-Li degree of polymerization.

We prepared 0.5 molal solutions of the STFSI-Li polymers in 30 : 70 wt% EC : EMC for electrochemical and transport measurement. We chose this concentration because 0.5 m is near the peak in conductivity for the STFSI-Li monomer solution (see Fig. 2) while the relatively low concentration minimizes polymer salt material use. Physical properties of the polyelectrolyte solutions are reported in Table 2. We observe a sharp decrease in conductivity upon oligomerization, with the 5 repeat unit polyelectrolyte solution exhibiting a conductivity of 1 mS cm^−1^, a 50% reduction in conductivity from the monomer despite containing the same concentration of ions (see Fig. 2). There is very minimal change in solution viscosity upon oligomerization, suggesting the decrease in conductivity is primarily due to decreased mobility of the larger polyanions. Conductivity continues to slowly decrease with increasing degrees of polymerization.

**Table 2:** Solution properties for polyelectrolytes in 30 : 70 wt% EC : EMC

Polymer	Molality (mol kg^−1^)	wt% salt	Density (g mL^−1^)	Molarity (mol L^−1^)	Viscosity (mPa s)
Monomer	0.5	13.8	1.1422 ± 0.0001	0.49	2.52 ± 0.06
5 repeats	0.5	16.6	1.1462 ± 0.0001	0.48	2.91 ± 0.03
10 repeats	0.1	3.5	1.1060 ± 0.0001	0.11	1.53 ± 0.01
10 repeats	0.2	6.7	1.1165 ± 0.0001	0.21	1.74 ± 0.02
10 repeats	0.5	15.3	1.1447 ± 0.0001	0.49	3.10 ± 0.02
10 repeats	1.0	26.5	1.1849 ± 0.0001	0.87	11.6 ± 0.28
15 repeats	0.5	14.8	1.1415 ± 0.0001	0.49	2.94 ± 0.03
20 repeats	0.5	14.6	1.1382 ± 0.0001	0.49	2.92 ± 0.05
40 repeats	0.5	14.2	1.1380 ± 0.0001	0.49	3.31 ± 0.05

### 3.2 Ideal *vs.* real solution transport properties

**Fig. 3:**
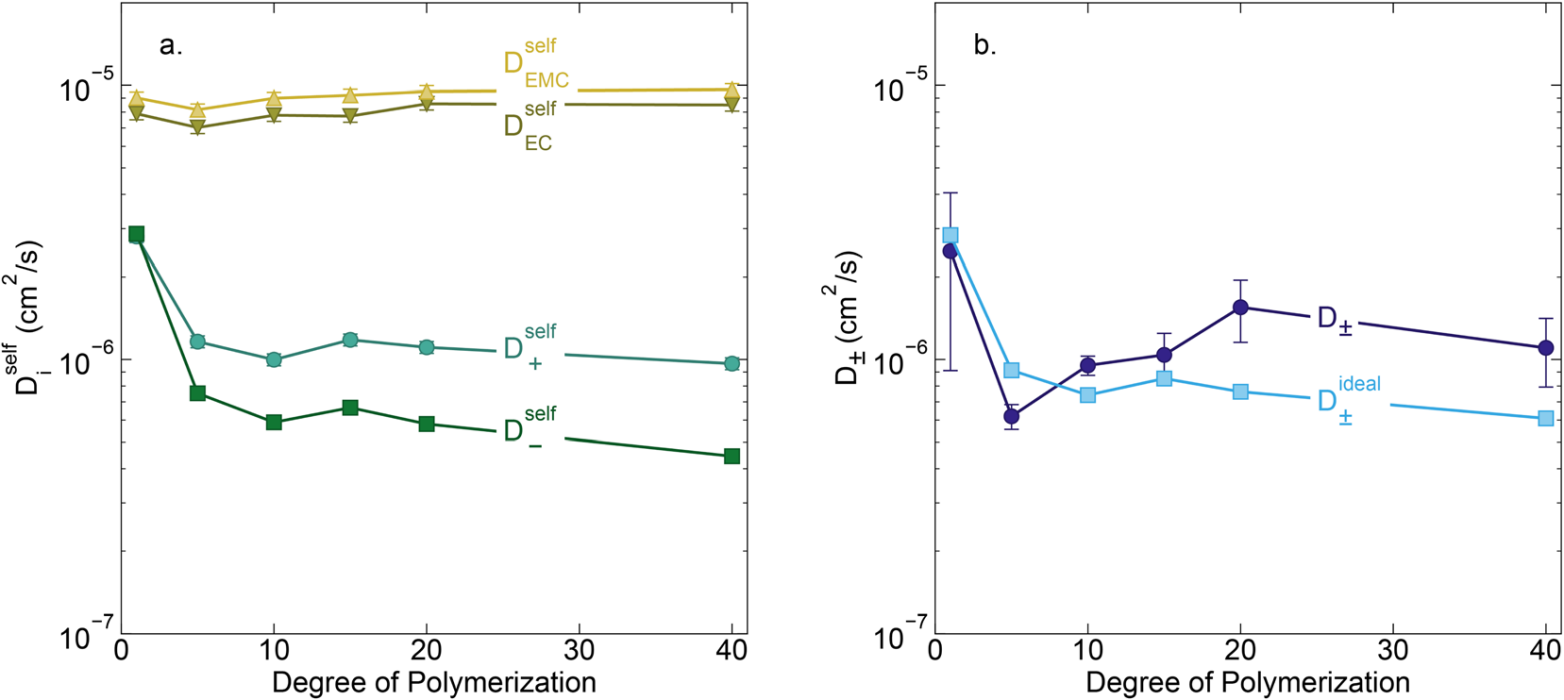
(a) Self diffusion coefficients (cm^2^ s^−1^) *vs.* degree of polymerization, as measured using PFG NMR, and (b) total salt diffusion coefficients (cm^2^ s^−1^) *vs.* degree of polymerization as measured using restricted diffusion (purple) and as calculated from PFG NMR results presented in panel a, assuming ideal solution behavior (cyan).

Conductivity data suggests slowing ion motion as the polyanion molecular weight increases. Self-diffusion coefficients of each species, as measured using PFG NMR, are reported in Fig. 3a. As expected, we see a decrease in both the cation and polyanion self diffusion coefficients with increasing polyanion chain length, with the anion self diffusion decreasing significantly more than that of the cation. For the 40 repeat unit polyanion, the anion self diffusion coefficient is reduced six fold compared to the monomer solution, from 2.9 × 10^−6^ to 4.4 × 10^−7^ cm^2^ s^−1^, whereas the lithium self diffusion coefficient decreases three fold, from 2.8 × 10^−6^ to 9.7 × 10^−7^ cm^2^ s^−1^. These trends are in alignment with previously reported polyelectrolyte solution systems.^5–7^

The Nernst–Hartley relationship allows us to calculate an ideal total salt diffusion coefficient (*D*_±_^ideal^) in the dilute solution limit from the harmonic mean of the cation and anion self diffusion coefficients.^35^*D*_±_^ideal^ assumes full dissociation of all ions and no explicit ion or solvent interactions. As expected, *D*_±_^ideal^ decrease steadily with increasing anion molecular weight (see Fig. 3b). Fig. 3b also shows the real solution total salt diffusion coefficients (*D*_±_) measured by the restricted diffusion method. We observe an initial decrease in *D*_±_ compared to the monomer for the 5 repeat unit oligomer followed by an unexpected increase in *D*_±_ for higher degrees of polymerization. One possible explanation for this phenomena is that with increasing polyanion molecular weight, positive distinct ion-correlations increase for all mobile ions. Positively correlated ionic motion would speed up overall salt transport.^36^ This type of behavior was recently observed by Sachar *et al.* in molecular dynamic simulations of salt transport in ligand functionalized polymer membranes where the thermodynamic factor was assumed to be equal to 1.^36^ Making the same assumption of a unity thermodynamic factor, we can calculate *D*_±_ from previously published molecular dynamic studies of PESs^11^ and again observe *D*_±_ increase with increasing polyanion length (see Fig. S3†). This supports the conclusion that positively correlated distinct anion–anion and cation–cation motion increases the diffusion coefficient. Another explanation for the increasing speed of overall salt transport with increasing polyanion molecular weight could be due to changes in the salt thermodynamic factor with molecular weight.

The importance of correlated motion in the polyelectrolyte solution system becomes more obvious when comparing transference numbers obtained from eNMR to those obtained using ideal Nernst–Einstein assumptions. The ideal solution cation transference number (*t*_+_^0 ideal^) can be expressed as8
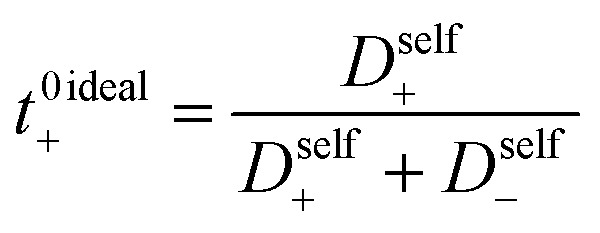
where the superscript ‘0’ represents the solvent frame of reference. *t*_+_^0 ideal^ does not account for any ion–ion or ion–solvent interactions, however PESs have inherent ion interactions through covalent bonding of anions to each other along the polymer chain. Therefore Fong *et al.* proposed the correction to the ideal transference number (*t*_+_^0 id,polymer^) in eqn (9) that accounts for inherent correlation of polymerized anions through the backbone while still ignoring cation–anion interactions, cation–cation interactions, and distinct anion interactions between ions on different polymer backbones9
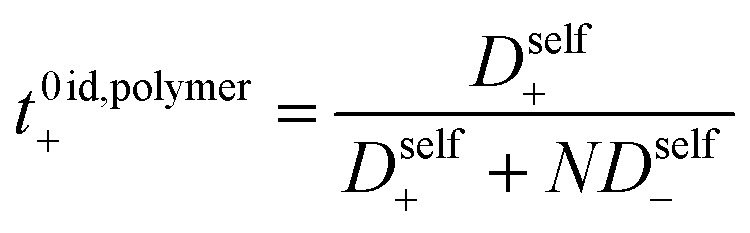
where *N* represents the polyanion degree of polymerization.^12^ Finally the true cation transference number (*t*_+_^0^), defined as the ratio of current carried by the cation to the total ionic current under conditions of no concentration gradients, can be calculated from electrophoretic mobilities according to eqn (10).10
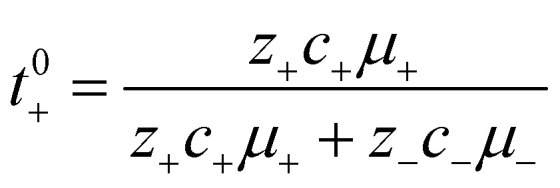


**Fig. 4:**
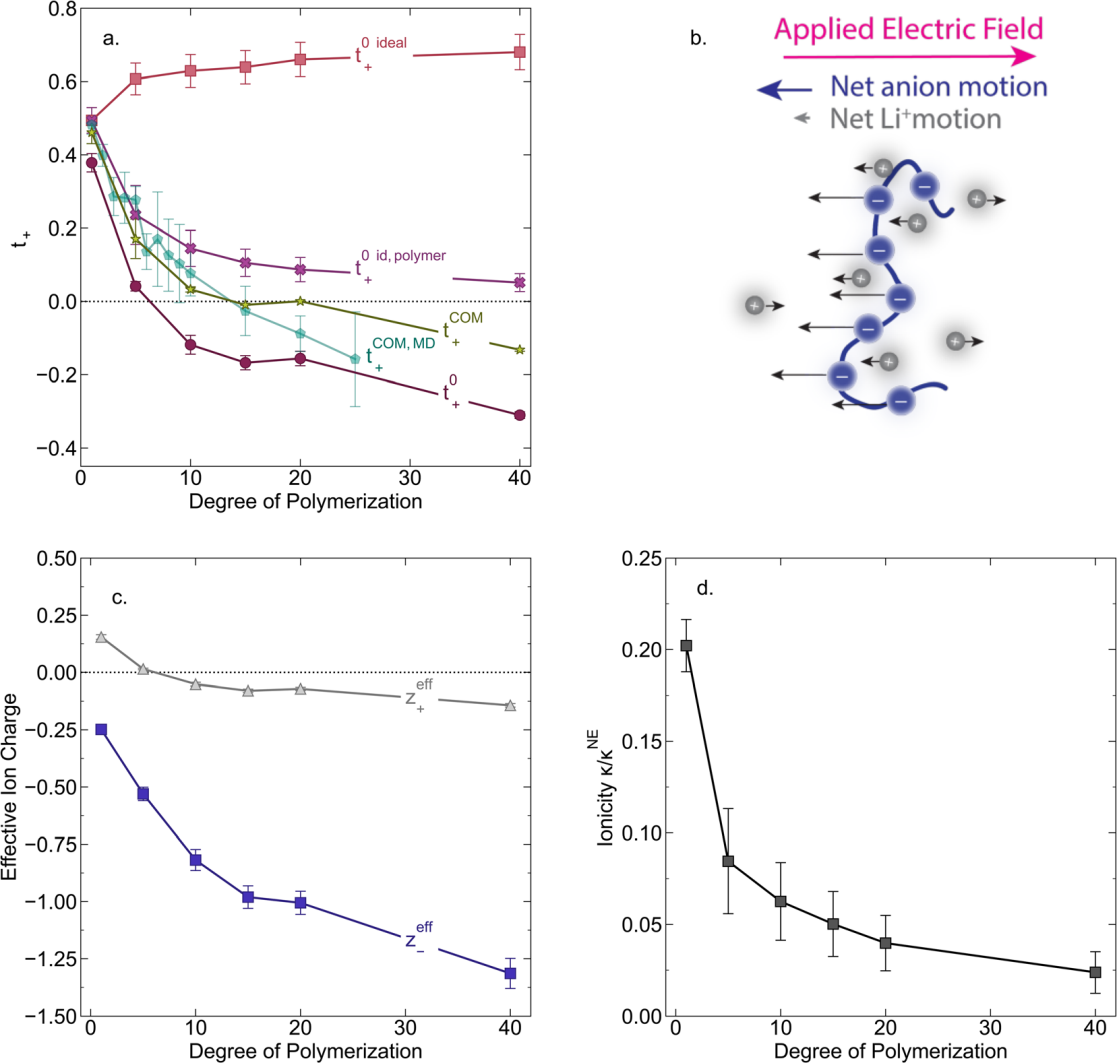
(a) Transference number *vs.* degree of polymerization calculated using ideal solution conditions (*t*_+_^0 ideal^), ideal polymer conditions (*t*_+_^0 id,polymer^), and rigorous real solution conditions (*t*_+_^0^, *t*_+_^COM^). *t*_+_^0 ideal^ and *t*_+_^0 id,polymer^ were calculated from PFG NMR measurements, while *t*_+_^0^ and *t*_+_^COM^ were calculated from eNMR measurements. *t*_+_^COM^ is reported to allow comparison with *t*_+_^COM,MD^ that was calculated from course-grained molecular dynamic simulations reproduced from ref. 11. (b) Schematic of conditions leading to a negative transference number. (c) Effective ion charge for the anion and cation. (d) Ionicity (*κ*/*κ*^NE^) *vs.* degree of polymerization. All experimental data corresponds to 0.5 m Li^+^ whereas MD simulation data presented here corresponds roughly to a cation concentration of 0.48 M.

In Fig. 4a we plot the transference numbers calculated according to eqn (8)–(10). Both *t*_+_^0^ and *t*_+_^COM^ are calculated from eNMR data however reflect a shift in reference frame from the solvent for the former and center of mass for the latter. Similar to prior literature we observe a highly favorable ideal transference number trend with *t*_+_^0 ideal^ increasing with degree of polymerization approaching 0.7 at the highest degrees of polymerization. However if we account for the much more realistic ideal polymer solution conditions that account for the multivalent nature of the polyanions but no other ion interactions, *t*_+_^0 id,polymer^ decreases exponentially with degree of polymerization. Similar to *t*_+_^0 id,polymer^, the true transference number shows a rapid decrease with increasing polyanion chain length. However, *t*_+_^0^ becomes negative between 5 and 10 degrees of polymerization. With reference to the center of mass, *t*_+_^COM^ becomes negative at 15 degrees of polymerization. A negative transference number implies that under the conditions of no concentration gradients, net lithium motion will be opposite the electric fields. This could occur if lithium primarily exists in negatively charged clusters, as would be the case for lithium ions condensed to the polyanionic chain. Fig. 4b illustrates such a scenario, where free disassociated lithium ions move in the “correct” direction with the electric field while net lithium motion is in the “wrong” direction with condensed lithium species dragged against the electric field with the polyanion. We directly observe this drift of lithium in the “wrong” direction in an electric field in our eNMR spectra that produce the same sign of phase shift for both lithium and polyanion peaks. While this phenomena has been directly observed in ionic liquid systems,^37–39^ and indirectly in solid polymer systems,^40,41^ we believe this is the first experimental observation of a negative transference number in a non-aqueous polyelectrolyte solution. The large negative shift in *t*_+_^0^ compared to *t*_+_^0 id,polymer^ highlights that in addition to intra-chain anion–anion correlation captured by *t*_+_^0 id,polymer^, cation–anion correlation is also significant in these systems. Additionally there is likely also positive cation–cation correlation between cations condensed on the same polymer chain.

By performing a force balance between the coulombic forces under an electric field and the hydrodynamic friction forces on ions during an eNMR experiment, we can calculate an effective ion charge (*z*_i_^eff^) according to eqn (11).^42–44^11
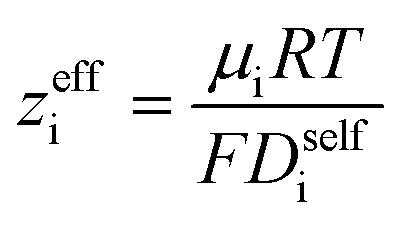


For a fully dissociated salt, we would expect the effective anion charge to be equal to the nominal charge (*z*_−_^eff^ = −*N*). In Fig. 4c, we plot the effective charge on the Li-ion and the polyanion. We observe that the effective charge on the polyanion is far lower than the nominal charge for all molecular weights, indicative of significant counter-ion condensation (cation–anion correlation). For the 40 repeat unit polyanion, only 3.3% of the nominal charge is effective (dissociated) compared to 25% for the monomer. We also observe that the effective charge on the Li^+^ decreases with molecular weight and becomes negative, consistent with our picture of negatively charged ion clusters in Fig. 4b.

Using the ion self diffusion coefficients and the Nernst–Einstein relationship, we can also calculate a theoretical ionic conductivity (*κ*^NE^) for an ideal solution with no ion interactions according to12
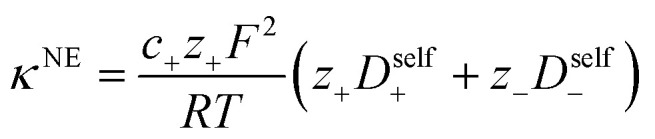
where *z*_−_ = −*N*, which accounts for polymeric nature of the polyanion chain. Accordingly we can calculate the ionicity (*κ*/*κ*^NE^) which is often interpreted as a measure of the extent of salt dissociation or the degree of uncorrelated ion motion. We observe the ionicity decreases as a function of chain length from 0.2 for the monomer to 0.024 at 40 repeat units. This implies that ion correlation reduces the theoretical solution conductivity by ∼80–98% in 0.5 m solutions (see Fig. 4c).

### 3.3 Quantifying ion correlation contributions *via* Onsager coefficients

In order to quantify the contributions of different ion-correlations to the overall solution conductivity, we can look at the Onsager transport coefficients, *L*^*ij*^. There are 3 independent Onsager coefficients for a binary system – *L*^+−^, *L*^++^, and *L*^−−^. *L*^+−^ captures the correlated motion between cation and anions, while *L*^++^ and *L*^−−^ capture the correlated motion between like charged particles. The transport coefficients *L*^*ii*^ can be separated into a self term *L*_self_^*ii*^ which accounts for ideal self-diffusion, and a distinct term *L*_dist_^*ii*^ which captures correlations between particles.^45–49^ We note that for the purposes of our analysis we formulate the Onsager coefficients from the perspective of an individual anion repeat unit (*z*_−_ = −1) as this is consistent with the conventional use of the Nernst–Einstein equation and allows for easy comparison between the approximated ideal Nernst–Einstein behavior and the more rigorous non-ideal transport behavior. This means that the Onsager term *L*_dist_^−−^ captures both intra-chain correlations through the covalent bonds of the polymer backbone and the inter-chain correlations between different polymer chains. We can easily convert between the monomeric formulation of the Onsager coefficient to one where we consider the anion as the full polyanion (*z*_−_ = −*N*) and *L*_dist_^−−^ captures only inter-chain correlations (see ESI†). Onsager transport coefficients can be calculated from experimental transport quantities (*t*_+_, *κ*, *D*_±_) according to eqn (13)–(17) if the thermodynamic factor, which relates gradients in concentration to gradients in electrochemical potential, is known.^49^13
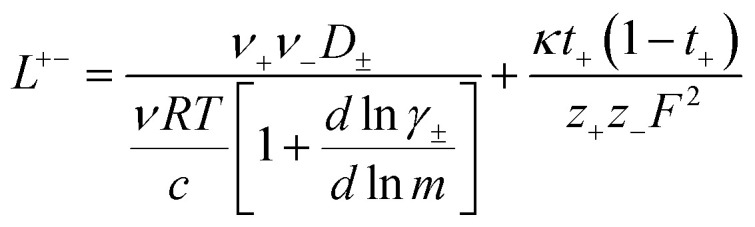
14
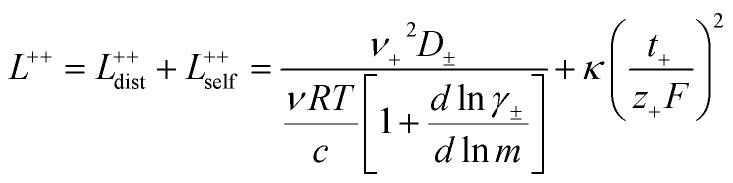
15
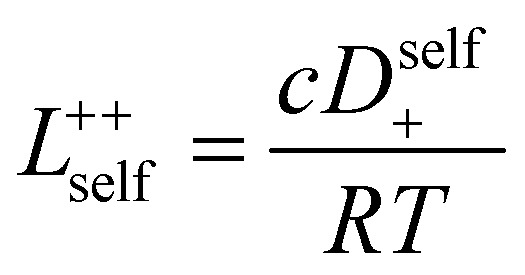
16
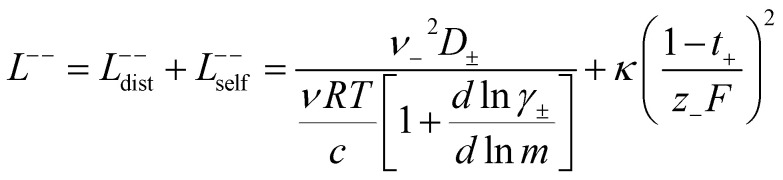
17
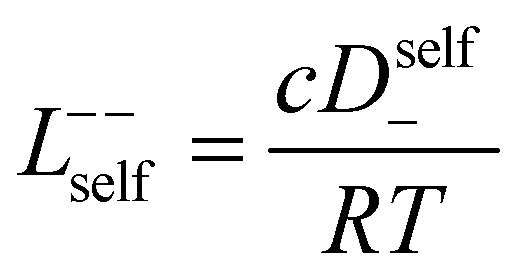


**Fig. 5:**
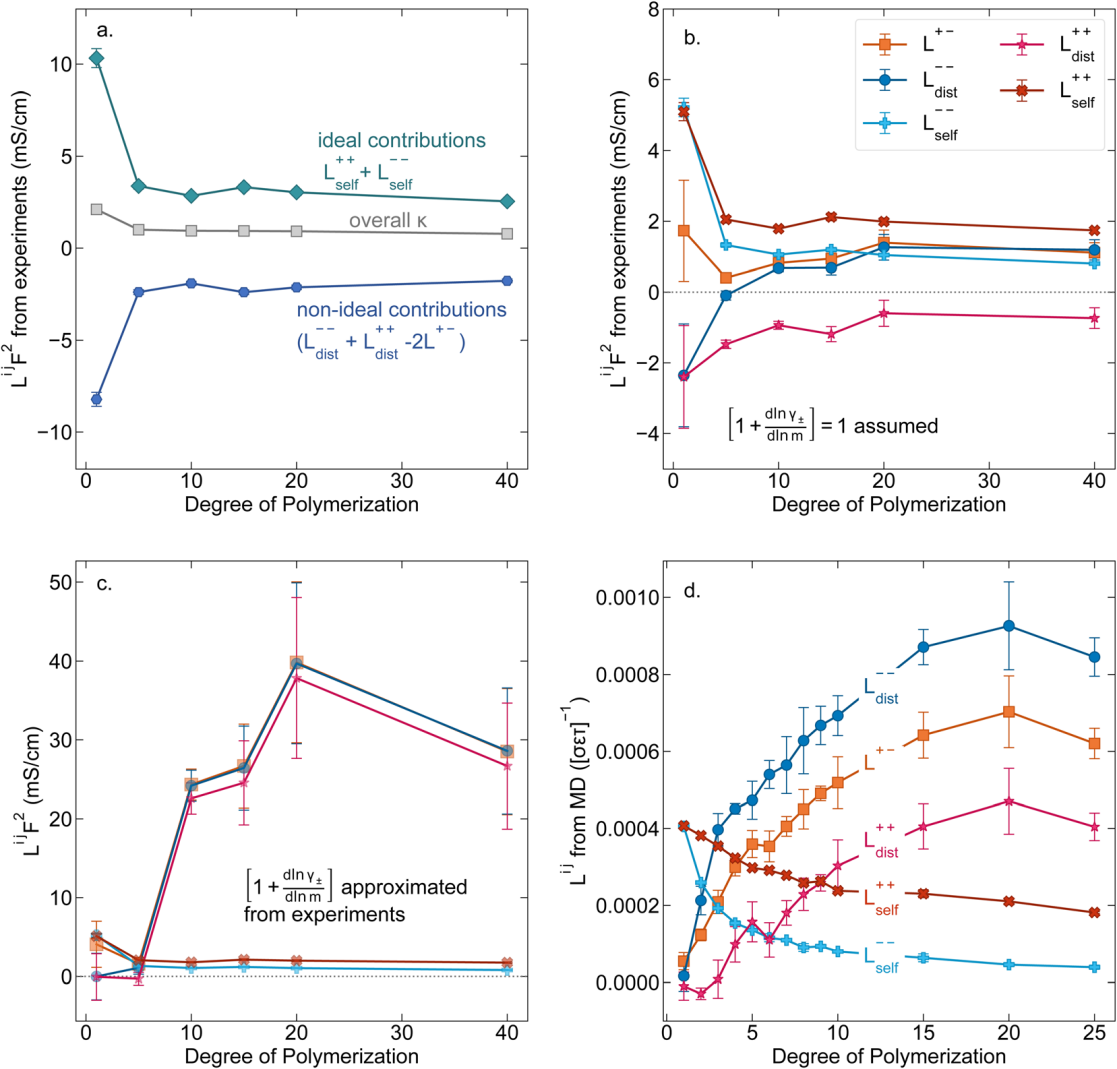
(a) Onsager transport coefficients broken into ideal [*L*_self_^++^ + *L*_self_^−−^] and non-ideal [*L*^++^_dist_ + *L*^−−^_dist_ − 2*L*^+−^] contributions to conductivity as calculated from experimental data (conductivity probe, eNMR, and restricted diffusion measurements) with no assumptions. (b) Onsager transport coefficients calculated from experimental data assuming that 
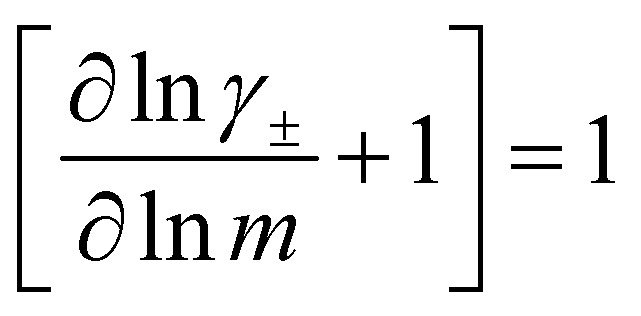
. (c) Onsager transport coefficients calculated using U-cell data for the thermodynamic factor. Note an approximate thermodynamic factor of 0.035 is used for polyanions with 10 or more repeat units, with thermodynamic factors for the monomer and 5 repeat unit polyanion given in Fig. S4b.† Legend is same as panel b. (d) Onsager transport coefficients *L*^*ij*^*vs.* degree of polymerization calculated with coarse grain molecular dynamics, reproduced from ref. 11. Note the molecular dynamics *L*^*ij*^ are all reported in Lennard Jones units corresponding to dimensions of (length × energy × time)^−1^. Simulation data presented here corresponds roughly to a cation concentration of 0.48 M.

While we attempted to measure the thermodynamic factor using methods well known in the literature and described in the methods section, the higher molecular weight polyelectrolyte solutions systems exhibit thermodynamic factors approaching zero – an unusual behavior which demand explanation outside the scope of this work (see ESI† for description of this behavior). Without the thermodynamic factor, we can still calculate self correlation Onsager terms, *L*_self_^++^ and *L*_self_^−−^, as well as the sum of distinct particle interactions captured by [*L*^++^_dist_ + *L*^−−^_dist_ − 2*L*^+−^] (see Fig. 5a). Corresponding to the decrease in self-diffusion coefficient we see that *L*_self_^−−^ and *L*_self_^++^ decrease with degree of polymerization. The term [*L*^++^_dist_ + *L*^−−^_dist_ − 2*L*^+−^] accounts for the total amount of correlated motion between distinct ions in solution. We observe that [*L*^++^_dist_ + *L*^−−^_dist_ − 2*L*^+−^] increases (becomes less negative) with increasing degree of polymerization, indicating either an increase in distinct anion–anion and cation–cation correlated motion or a decrease in correlated cation–anion motion. That the sum [*L*^++^_dist_ + *L*^−−^_dist_ − 2*L*^+−^] is negative indicates that strong cation–anion correlation (ion-pairing) exists across all degrees of polymerization. If we assume that the salt activity coefficient does not change significantly with concentration and therefore the thermodynamic factor is unity, we can also calculate *L*^*ij*^ for each polymer solution and therefore the relative contributions of *L*_dist_^++^, *L*_dist_^−−^, and *L*^+−^. We note that this ideal thermodynamic factor assumption is certainly not rigorous for these systems, nevertheless, the trends observed in *L*^*ij*^ are instructive. In Fig. 5b we observe that indeed both *L*_dist_^++^ and *L*_dist_^−−^ increase, with anion–anion correlated motion changing most strongly with degree of polymerization. *L*^+−^ also increases slightly with chain length, again indicating more ion-pairing for longer polyanion systems. This trend also holds if we use the thermodynamic factor obtained from U-cell measurements where we approximate the thermodynamic factor to be 0.035 for polymers 10 repeat units or longer (see Fig. 5c). This approximation is used to avoid divergence of *L*^*ij*^ given that we measure a small negative thermodynamic factor for the 40 repeat unit polymer (see Fig. S4†). The value 0.035 is the average measured thermodynamic factor for the 10, 15, and 20 repeat unit polyelectrolyte solutions (see Fig. S4b†). Again in Fig. 5c we see *L*_dist_^++^, *L*_dist_^−−^, and *L*^+−^ increase with degree of polymerization with the contributions with *L*_dist_^−−^, and *L*^+−^ having the largest contributions. These observations together corroborate that the increase in ion-interactions captured by the term [*L*^++^_dist_ + *L*^−−^_dist_ − 2*L*^+−^] is due primarily to increase in distinct anion–anion correlated motion.

We further examine Onsager coefficients calculated from course-grained molecular dynamics (MD) previously calculated by our group to confirm the relative magnitude of different correlations. All molecular dynamic results are reproduced from ref. 11 with permission of the publisher. We are confident in the applicability of this model due to excellent agreement in *t*_+_ between our experimentally measured and the non-chemically specific MD model (see Fig. 4a). Our experimental observations of decreasing conductivity with chain length, despite little increase in solution viscosity, are consistent with MD findings that *L*^+−^ is positive and increases with increasing chain length, an indication that ion-pairing becomes more prevalent with increasing chain length.^11^ Under the conventional picture of fully dissociated ions in solution, we would expect like-charged particles to repel, resulting in negative *L*_dist_^++^ and *L*_dist_^−−^ terms. As discussed previously this picture is not realistic at least for the polyanion where anions on the same chain must be positively correlated. We found that correcting ideal assumptions to account for intra-chain anion correlations and no other non-idealities as in eqn (9) is sufficient to reproduce the shape of the *t*_+_*vs.* chain length trend shown in Fig. 4a. This indicates that intra-chain anion correlation is likely the largest factor influencing the decrease in *t*_+_ with chain length. This theory is again supported by MD which shows that *L*_dist_^++^ and *L*_dist_^−−^ become increasingly positive with *L*_dist_^−−^ having the largest contribution.^11^ The increase and positive sign of *L*_dist_^++^ with increasing chain length shown in MD simulations are supportive of our picture of multiple cations condensed to the same chain moving as an ion cluster.^11^ Combined with increased ion pairing (increasing positive *L*^+−^), this ultimately results in the negative transference number observed in experiments. We can confirm that the large positive contribution of *L*_dist_^−−^ is almost entirely due to anion correlation through covalent bonds on a given chain by examining the Onsager coefficients where the anions are taken to be the entire polymer chains rather than individual monomers (*z*_−_ = −*N* rather than *z*_−_ = −1). In polyanion formulation of Onsager coefficients (denoted here with a subscript p), *L*_p,dist_^−−^ captures only inter-chain correlations like chain repulsion or chain aggregation. *L*_p,dist_^−−^ is near zero across all studied molecular weights indicating there is very little correlation between the motion of different polyanion chains (see Fig. S5†).

### 3.4 Concentration effects

**Fig. 6:**
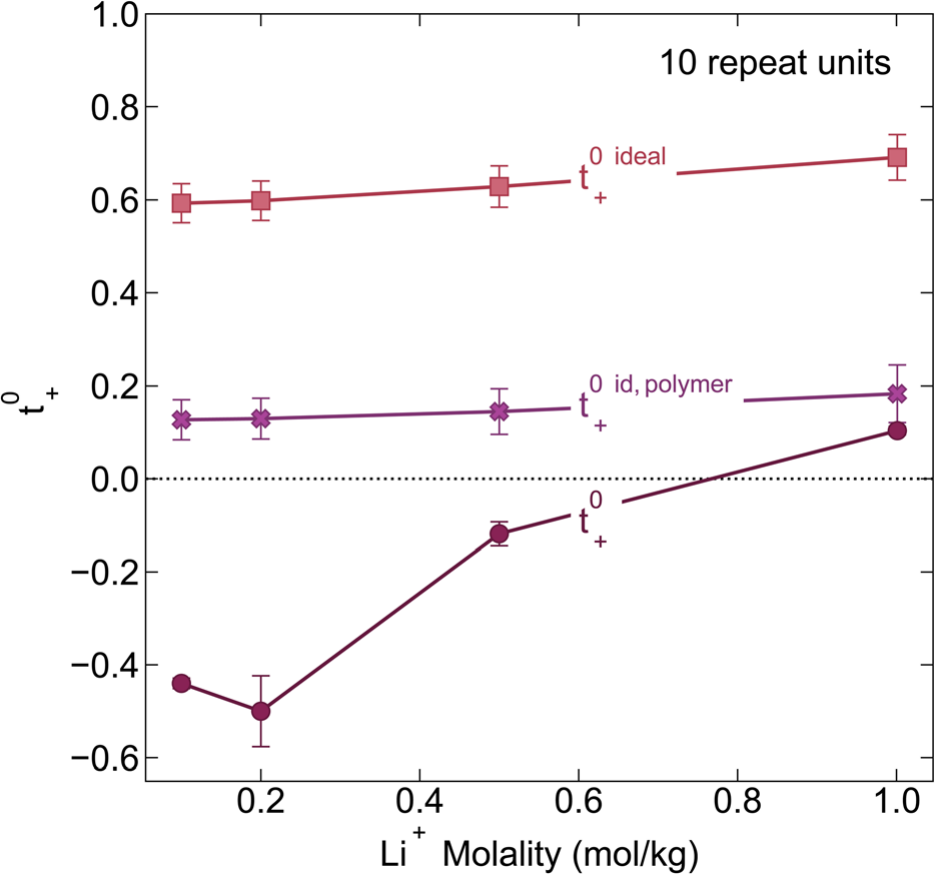
Transference number *vs.* lithium molality for polyelectrolyte solutions containing the 10-repeat unit polyanion. Once again the transference number is reported as calculated according to ideal solution conditions (*t*_+_^0 ideal^), ideal polymer conditions (*t*_+_^0 id,polymer^), and rigorous real solution conditions (*t*_+_^0^).

We chose the 10 repeat unit PSTFSI-Li system to study the effects of concentration on ion-correlation and transference number. We observe that *t*_+_ increases with increasing concentration, transitioning from a negative transference number below 0.5 m to slightly positive at 1.0 m (see Fig. 6). This observation is consistent with molecular dynamic studies^11,12^ and experimental studies of aqueous polyelectrolyte solutions.^50–52^ Both *t*_+_^ideal^ and *t*_+_^id,polymer^, as measured using self diffusion coefficients from PFG NMR, also show a slight increase with increasing concentration, however, neither capture the magnitude of the actual *t*_+_ increase or the transition from negative to positive values at higher concentrations. This phenomena can be primarily attributed to a decrease in the relative per-ion contribution of cation–anion correlations (*L*^+−^/*c*) with increasing concentration.^11^ Because neither *t*_+_^ideal^ nor *t*_+_^id,polymer^ capture cation–anion correlation, they are unable to capture these concentration effects. While the fraction of cations in ion pairs or multi-ion clusters generally increases with concentration, Fong *et al.* showed that the lifetime of these ion pairs decreases with concentration.^11^ At low concentrations there are few but long-lived ion pairs that contribute to more correlated ion-motion than at high concentration where there are more but shorter-lived ion pairs. In other words, in long-lived pairs lithium ions experience more vehicular-type motion, whereas in short-lived ion pairs, lithium ions hop between different anionic coordination environments. Negative lithium transference numbers at low concentrations are consistent with this picture as lithium ions bound to negatively charged clusters for longer periods of time will move with the polyanion in an applied electric field.

## 4 Conclusion

Five lithium-bearing oligomeric polyelectrolytes with controlled degrees of polymerization from 5 to 40 repeat units were synthesized. The transport properties of the monomer and polyelectrolytes were studied in EC : EMC solutions using a variety of experimental methods that allowed us to evaluate the contributions of different ion-correlations to the overall ionic motion as a function of polyanion molecular weight and concentration. Electrophoretic NMR measurements showed that the true transference number decreases with increasing polyanion molecular weight, with the transference number eventually becoming negative, indicative of significant ion clustering. PFG NMR experiments, along with insight from previously published MD results, demonstrate that this trend is primarily due to correlated intra-chain anion motion as indicated by a large, positive *L*_dist_^−−^ and to significant cation–anion correlation. We further show that the true transference number increases with increasing concentration and at a given polyion molecular weight the transference number can transition from negative to positive. Again, prior MD simulations show that these concentration effects are due to shorter-lived ion pairs at higher concentrations, reducing the overall contribution of cation–anion correlation. Chelating additives such as crown ethers that improve ion dissociation may help preserve positive transference numbers for polyelectrolyte solutions^39,53,54^ but due to strong intra-chain anion–anion correlation that is unaffected by these additives, we would expect the monomeric salt to remain the highest conductivity and highest *t*_+_ electrolyte. We note that this study was limited to the unentangled polymer regime and it is possible that highly entangled gel-like polyelectrolyte solutions where the polyanion is effectively immobilized could display different behavior. In confirmation of prior MD simulations, we find that polyelectrolyte solutions experience significant ion correlation through the polymer backbone and this non-ideality results in a strong deviation between the true *t*_+_ and the ideal solution *t*_+_^ideal^ behavior.^11,12^ Unlike all previous experimental studies on non-aqueous PESs,^3–5,7^ this leads us to conclude that short-chain polyelectrolyte solutions are not viable high *t*_+_, high conductivity electrolytes. This study again highlights the pitfalls of using experimental methodology that rely on ideal solution assumptions for battery electrolyte engineering.

## Data availability

Data used in figure production is available at https://doi.org/10.6078/D1PX3X. Further data is available upon reasonable request.

## Author contributions

H. K. Bergstrom: conceptualization, methodology, validation, formal analysis, investigation, visualization, writing – original draft. K. D. Fong: conceptualization, writing – review & editing. D. M. Halat: methodology, validation, writing – review & editing. C. A. Karouta: methodology, validation, writing – review & editing. H. C. Celik: resources, supervision, writing – review & editing. J. A. Reimer: resources, supervision, writing – review & editing. B. M. McCloskey: conceptualization, supervision, funding acquisition, writing – review & editing.

## Conflicts of interest

There are no conflicts to declare.

## Supplementary Material

SC-014-D3SC01224G-s001
