# Supplementary material for: Ion correlation and negative lithium transference in polyelectrolyte solutions
Source: Chem Sci. 2023 May 16;14(24):6546–57. doi: 10.1039/d3sc01224g (PMC10283486; doi:10.1039/d3sc01224g)
Supplement: SC-014-D3SC01224G-s001 [file SC-014-D3SC01224G-s001.pdf]

Electronic Supplementary Material (ESI) for Chemical Science.
This journal is © The Royal Society of Chemistry 2023

Ion Correlation and Negative Lithium Transference in Polyelectrolyte Solutions
Supplementary Information

Helen K. Bergstrom,[1,2‡] Kara D. Fong,[1,2] David M. Halat,[1,3] Carl A. Karouta,[1,3] Hasan C. Celik,[4] Jeffrey A. Reimer,[1,3] Bryan D. McCloskey[1,2§]

[1] Department of Chemical & Biomolecular Engineering, University of California, Berkeley, CA 94720, USA

[2] Energy Technologies Area, Lawrence Berkeley National Laboratory, Berkeley, CA 94720, USA

[3] Materials Sciences Division and Joint Center for Energy Storage Research, Lawrence Berkeley National Laboratory, Berkeley, CA 94720, USA

[4] College of Chemistry NMR Facility, University of California, Berkeley, CA, 94720, USA

‡ helen_bergstrom@berkeley.edu

§ bmcclosk@berkeley.edu

1 RAFT Polymerization & End-Group Removal

Originally, RAFT polymerization was chosen as the preferred method for poylelectrolyte synthesis due to it's tight control over molecular weight distributions and adaptability to a number of classes of monomers. In order to limit the size and therefore effects of end-groups 2-cyano-2-propyl benzodithioate was chosen as the chain transfer agent. Oligomers were prepared by loading STFSILi monomer and the appropriate amount of RAFT agent for the desired DP into a round bottom flask and dissolved in anhydrous DMF inside an argon filled glovebox. Once the RAFT agent and monomer solution was fully dissolved, a solution of AIBN in DMF (5:1 molar ratio RAFT Agent: AIBN) was added before capping the flask with a rubber septa and removing it from the box. Oligomerization reactions were carried out at 75°C in an oilbath for 12 - 15 hours after which time, percent conversion appeared to no longer increase.

For oligomers over 15 repeat units long, these conditions were sufficient to achieve >95% conversion. However, for oligomers smaller than 15 repeat units (in this study 5 and 10 repeats), conversions were limited to <75 % even with extended reaction times and elevated temperatures. Dithiobenzoate groups are known to retard polymerization by cross-termination of very short radicals at high concentrations necessary to achieve very short oligomers. [1, 2] In order to overcome this limitation we selected a trithiocarbonate RAFT agent, 2-(Dodecylthiocarbonothioylthio)-2-methylpropionic acid, which is less likely to cause retardation, however introduces an additional challenge that the resulting large dodecyl end group could introduce significant end-group effects. [1] We therefore attempted to remove the RAFT agent end group and replace it with a less bulk moiety. A number of techniques from the literature were attempted without success including direct radical induced reduction with AIBN and lauroyl peroxide [3, 4], aminolysis followed by Michael addition thiol-ene reaction [5, 6], and Eosin-Y catalyzed UV trithiocarbonate cleavage. [7] For the radical induced reduction route, we observed incomplete cleavage of the trithiocarbonate group via UV-Vis. For the aminolysis-based methods, we were unable to drive complete aminolysis of the trithiocarbonate into the corresponding urea, and instead observed dithiocarbamate formation as evidenced by a color change of the polymer from yellow to pink.

2 Pulsed Field Gradient NMR

Representative values for diffusion pulse sequence and gradient parameters used in pulsed field gradient (PFG) NMR diffusion experiments are listed in Table S1. Here δ is the gradient pulse duration and Δ is the drift delay. For all experiments a repetition time of 5 seconds was used. For each nucleus 16 linearly spaced gradient steps starting at 5% of the maximum gradient strength were acquired.

Table S1: Representative PFG NMR parameter values

Nucelus	δ (msec)	Δ (msec)	Max Gradient Strength (T/m)
^{7}Li	2.0	50	275-400
^{19}F	2.0	50	150-200
^{1}H	1.0-2.0	20-50	150-250

3 Electrophoretic NMR

Representative values for gradient and voltage parameters used in electrphoretic (eNMR) experiments are listed in Table S2. Gradient parameters were chosen such that the peak of interest was attenuated by $\sim$75%. The lower end of the applied voltage range was selected such that a phase shift was discernible, $\sim 1°$, while the upper voltage range was selected as the highest voltage before signal attenuation due to convection was observed. Exemplary phase shift data vs. $g \cdot V \cdot \delta \cdot L^{-1}$ for each nucleus is presented in Fig. S1 where g is the gradient strength in Tesla per meter, V is the applied voltage in volts, δ is the drift time in seconds, and L is the electrode separation distance in meters. The reported ^{7}Li, ^{19}F, and ^{1}H data correspond to lithium ions, polyanion, and Ethyl Methyl Carbonate (EMC) respectively. Note that the slope of the phase shift vs. $g \cdot V \cdot \delta \cdot L^{-1}$ is positive for both fluorine and lithium indicating that for this sample in net, lithium and fluorine velocities are in the same direction. There is no clear systematic phase shift for ^{1}H data indicating that within error the velocity of EMC molecules is 0 m s^{-1}V^{-1}. For all polyanion chain lengths we found that the mobility of both EC and EMC are 0 m^{2}s^{-1}V^{-1} within measurement error.

Table S2: Representative eNMR parameter values

Nucelus	Gradient Strength (T/m)	Applied Voltage (V)
^{7}Li	1.5-2.5	75-250
^{19}F	0.5-0.8	25-170
^{1}H	0.25-0.4	100-300

Convection-based artifacts and spurious phase shifts can be significant issues in eNMR measurements. These artifacts can arise from a number of sources including Joule heating, electroosmotic flow, bubble formation, and physical movement of electrodes and wires under an applied magnetic gradients and electric fields. To address bubble formation particularly from hydrogen evolution, we use Palladium electrodes which are known to readily dissociate and absorb hydrogen gas. [8] To address concerns of convection due to Joule heating and electroosmotic flow we use a multi-pronged approach. First, we use a truncated CPMG-like the pulse sequence designed specifically to suppress convective artifacts. [8–10] The CPMG-like experiment used herein rapidly reverses the polarity of the applied electric field pulses halfway through the pulse sequence. We refer readers to the work of Hallberg et al. for a more detailed discussion and systematic study of the effects of eNMR pulse sequences on obtained phase data. [8] To reduce the risk of significant joule heating driven

natural convection, we wait 90-120 seconds between pulses to allow dissipation of any localized heating. While the CPMG-like pulse sequence should reduce the appearance of artifacts from electroosmotic or natural convective flow, prior to data collection we optimize voltage and gradient parameters for each sample to minimize convective phenomena in general. In reality, this is done by varying experimental parameters- in particular the maximum applied voltage at a fixed gradient strength to see the threshold at which we start to see significant (10%) signal attenuation due to diffusion. Voltage-dependent signal attenuation is a clear sign of convective artifacts as is non-linear phase shifts as a function of $g \cdot V \cdot \delta \cdot L^{-1}$ $(T \cdot s \cdot V \cdot m^{-2})$. Lastly to catch any systematic spurious phase shifts that do arise, we duplicated each experiment with positive and negative gradient encoding which should result in equal magnitude, but opposite sign phase shifts as a function of applied voltage. As described in the main text, in order to confirm that artifacts were minimized in our measurements we compared conductivity calculated from the obtained ion mobilities to those measured via impedance and conductivity probes. In Fig. S2 we plot a comparison of the calculated and measured conductivites. We see good overall agreement within error between the two methods, confirming our confidence in the eNMR measurements.

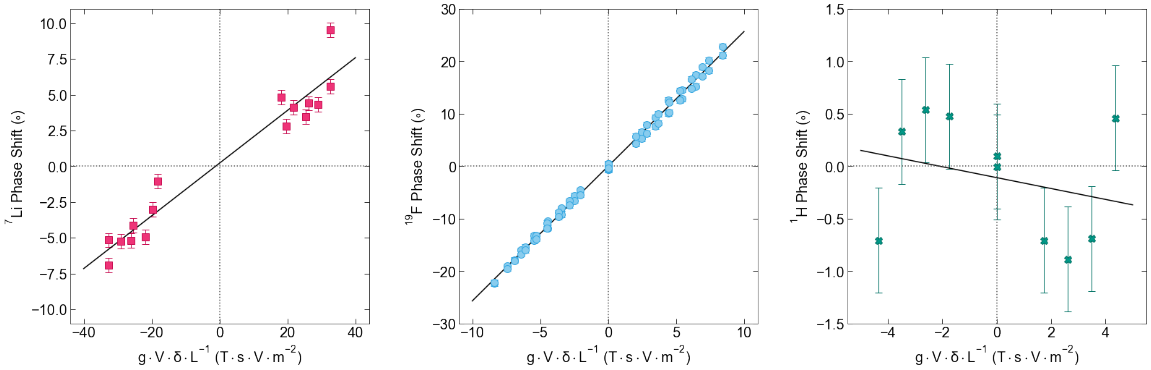

Figure S1: Phase shift (degrees) vs. $g \cdot V \cdot \delta \cdot L^{-1}$ $(T \cdot s \cdot V \cdot m^{-2})$ for three studied nuclei of 0.5m 10 repeat unit PSTFSI-Li in 3:7 EC:EMC. ^{7}Li points correspond to lithium ions in solution and condensed to the backbone, ^{19}F points correspond to the polyanion, and ^{1}H points shown here correspond to EMC molecules.

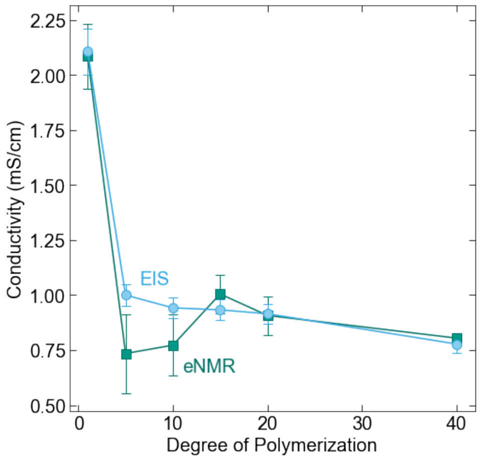

Figure S2: Conductivity (mS/cm) vs Degree of Polymerization as calculated from eNMR mobilities and measured by electrochemical impedance spectroscopy.

4 Diffusion Coefficients from MD

In the main text, we hypothesize that an increase in the total salt diffusion coefficient ($D_{\pm}$) with increasing molecular weight is due to increasing positive distinct ion correlations for all mobile ions. To support our hypothesis, we used previously published molecular dynamics data for polyelectrolyte solutions from Fong et al. and calculate $D_{\pm}$ from reported Onsager coefficients according to Eq. S1 (see Fig. S3). [11,12] Similar to the analysis by Sachar et al., we assume that the thermodynamic factor ($\left[1+\frac{dln\gamma_{\pm}}{d\ln m}\right]$) is unity. [13]

$$D_{\pm} = \frac{-z_+z_-\left(L^{--}L^{++}-L^{+-^2}\right)}{\nu_+\nu_-\left(z_+^2L^{++}+2z_+z_-L^{+-}+z_-^2L^{--}\right)}\frac{\nu RT}{c}(1-t_+)\left[1+\frac{\partial\ln\gamma_{\pm}}{\partial\ln m}\right] \tag{S1}$$

We observe that $D_{\pm}$ increases with increasing polyanion length which corresponds to an increase in positive distinct cation-cation, anion-anion and cation-anion correlations. We observe that $D_{\pm}$ increases with increasing polyanion length which corresponds to an increase in positive distinct cation-cation, anion-anion and cation-anion correlations. These findings are in line with Sachar et al.'s previous molecular dynamic simulations of salt transport in ligand functionalized polymer membranes where the thermodynamic factor was assumed to be equal to 1. [13] Further, Sachar proved that $\frac{\partial D_{\pm}}{\partial L^{++}_{dist}}$ and $\frac{\partial D_{\pm}}{\partial L^{--}_{dist}}$ are always positive for a single salt system. [13]

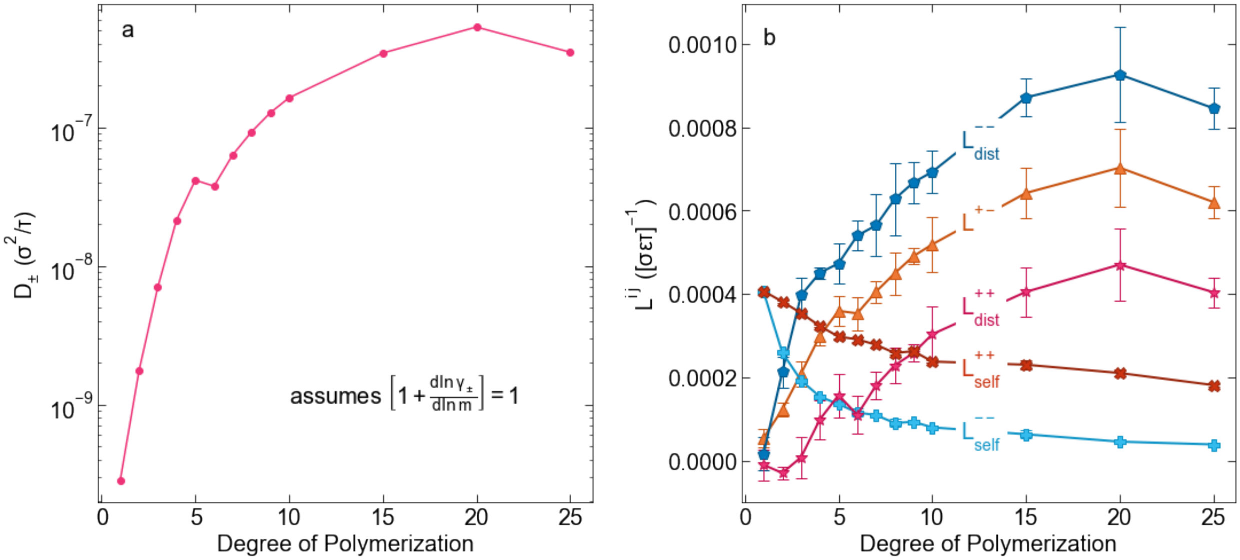

Figure S3: a) Total Salt Diffusion Coefficient vs. Degree of Polymerization as calculated from L^{ij} data from Fong et al. according to Eq. S1 assuming a thermodynamic factor of 1. [11] Note that diffusion coefficient is reported in Lennard Jones units of $\sigma^2\tau^{-1}$ (length^2 * time^{-1}). b) Onsager transport coefficients L^{ij} vs. degree of polymerization calculated with coarse grain molecular dynamics, reproduced from Ref. [11]. Note the L^{ij} are all reported in Lennard Jones units of $\sigma\epsilon\tau$ (length * energy * time)$^{-1}$. Simulation data presented here corresponds roughly to a cation concentration of 0.48M.

5 Thermodynamic Factor Measurements

As described in the main text, the change in liquid junction potential (U) across a concentration cell as a function of concentration is related to the transference number and thermodynamic factor according to

$$\frac{\partial U}{\partial \ln m} = \frac{\nu}{z_+\nu_+}\frac{RT}{F}(1-t_+)\left[1+\frac{\partial \ln \gamma_\pm}{\partial \ln m}\right] \tag{S2}$$

where m is the solution molality, ν_i is the stoicheometric coefficient of species i, and $\gamma_\pm$ is the molal activity coefficient. [14, 15] Concentration cell measurements were performed as described in the main text with a constant reference concentration of 0.1 mol/kg. We observed for polyanions over 5 repeat units long, liquid junction potentials are below 5 mV across an order of magnitude difference in concentration (see Fig. S4a. Typically we would expect liquid junction potentials on the order of 20 mV or greater according to the Nernst equation. Following Eq. S2 this leads to a thermodynamic factor that approaches zero (see Fig. S4b). A thermodynamic factor of 0 would imply that $\frac{\partial \ln \gamma_\pm}{\partial \ln m} \approx -1$ meaning the total salt activity coefficient is proportional to $\frac{1}{m}$. Put otherwise this means that for high molecular weights, the solution activity is not a function of salt concentration such that the effective concentration of polyelectrolyte in solution does not change even upon increasing the actual polyelectrolyte concentration.

$$\begin{aligned} L^{++} &= \frac{\nu_+^2 D_\pm}{\frac{\nu RT}{c}\left[1+\frac{d\ln\gamma_\pm}{d\ln m}\right]} + \kappa\left(\frac{t_+}{z_+F}\right)^2 \\ L^{--} &= \frac{\nu_-^2 D_\pm}{\frac{\nu RT}{c}\left[1+\frac{d\ln\gamma_\pm}{d\ln m}\right]} + \kappa\left(\frac{1-t_+}{z_-F}\right)^2 \\ L^{+-} &= \frac{\nu_+\nu_- D_\pm}{\frac{\nu RT}{c}\left[1+\frac{d\ln\gamma_\pm}{d\ln m}\right]} + \frac{\kappa\, t_+\,(1-t_+)}{z_+z_-F^2} \end{aligned} \tag{S3}$$

If the thermodynamic factor is known in addition to the electrolyte conductivity, the total salt diffusion coefficient, and the cation transference number, the Onsager transport coefficients can be calculated according to Eq. S3 [12]. Unfortunately, a thermodynamic factor approaching 0 leads to a divergence in Onsager transport coefficients, and small errors in the measured thermodynamic factor lead to massive uncertainty in L^{ij}. This is illustrated in Fig. S4c where we see the standard error for L^{++}_{dist}, L^{+-}_{dist}, and L^{+-} for the 40 repeat units polyelectrolyte solution is on the order of 2,000 mS/cm. The second law of thermodynamics requires that L^{++} and L^{--} are positive - a condition that is violated over the majority of the confidence interval for the 40 repeat unit polyelectrolyte solution [11, 12]. This region is shaded in grey in Fig. S4c.

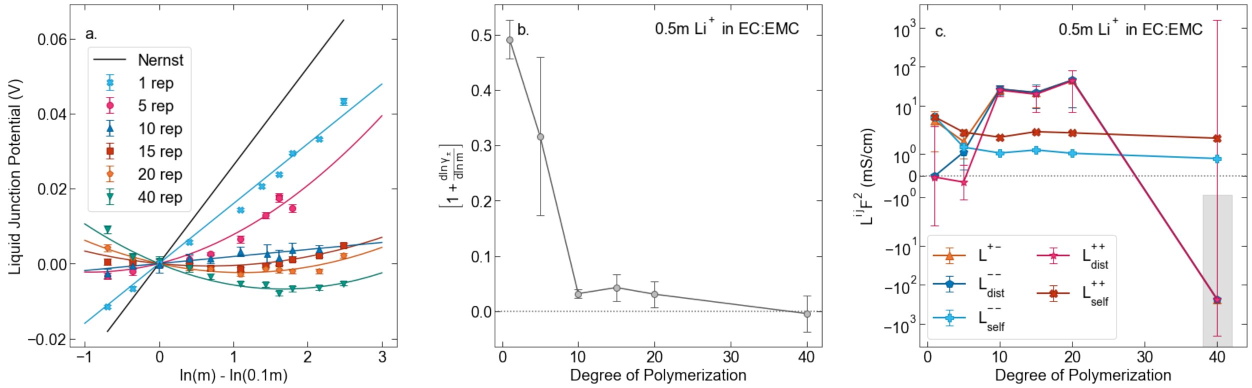

Figure S4: a) Liquid Junction Potential (V) vs. log of molality difference. b) Thermodynamic Factor vs. Degree of Polymerization at 0.5m Li^+ c) Onsager Transport Coefficients vs. Degree of Polymerization 0.5m Li^+ calculated using experimental data including the experimentally obtained Thermodynamic Factor. The gray box denotes values of L^{++}_{dist} and L^{--}_{dist} which would violate the second law of thermodynamics.

6 Onsager Coefficients for the Anion as a Full Polymer Chain vs. as a Monomeric Repeat Unit

As noted in the main text, for a polyion the Onsager transport coefficients L^{ij} can be formulated with respect to the individual ion repeat unit ($z_- = -1$) which we will denote with a subscript m or with respect to the entire polyanion ($z_- = -N$) denoted here with a subscript p. Both of these formulations are equally valid and can be inter-converted between according to equations S4. [11]

$$\begin{aligned} L^{--}_{\mathrm{p}} &= \frac{1}{N^2} L^{--}_{\mathrm{m}} \\ L^{--}_{\mathrm{p,self}} &= \frac{1}{N} L^{--}_{\mathrm{m,self}} \\ L^{--}_{\mathrm{p,dist}} &= \frac{1}{N^2} L^{--}_{\mathrm{m}} - \frac{1}{N} L^{--}_{\mathrm{m,self}} \\ L^{+-}_{\mathrm{p}} &= \frac{1}{N} L^{+-}_{\mathrm{m}} \end{aligned} \tag{S4}$$

Each formulation has its own advantage in terms of ease of interpretation. $L^{--}_{\mathrm{m,dist}}$ captures both intra-chain correlations through the covalent bonds of the polymer backbone and the inter-chain correlations between different polymer chains, whereas $L^{--}_{\mathrm{p,dist}}$ captures only inter-chain correlations. Comparing $L^{--}_{\mathrm{m,dist}}$ to $L^{--}_{\mathrm{p,dist}}$ allows one to separate the amount of distinct correlation due to covalent bonds inherent to polyions from correlation between separate chains. While $L^{--}_{\mathrm{p,dist}}$ provides a more straightforward picture of polyion correlated motion, L^{+-}_{p} is slightly more difficult to interpret as the cation and anion no longer have the same magnitude of charge.

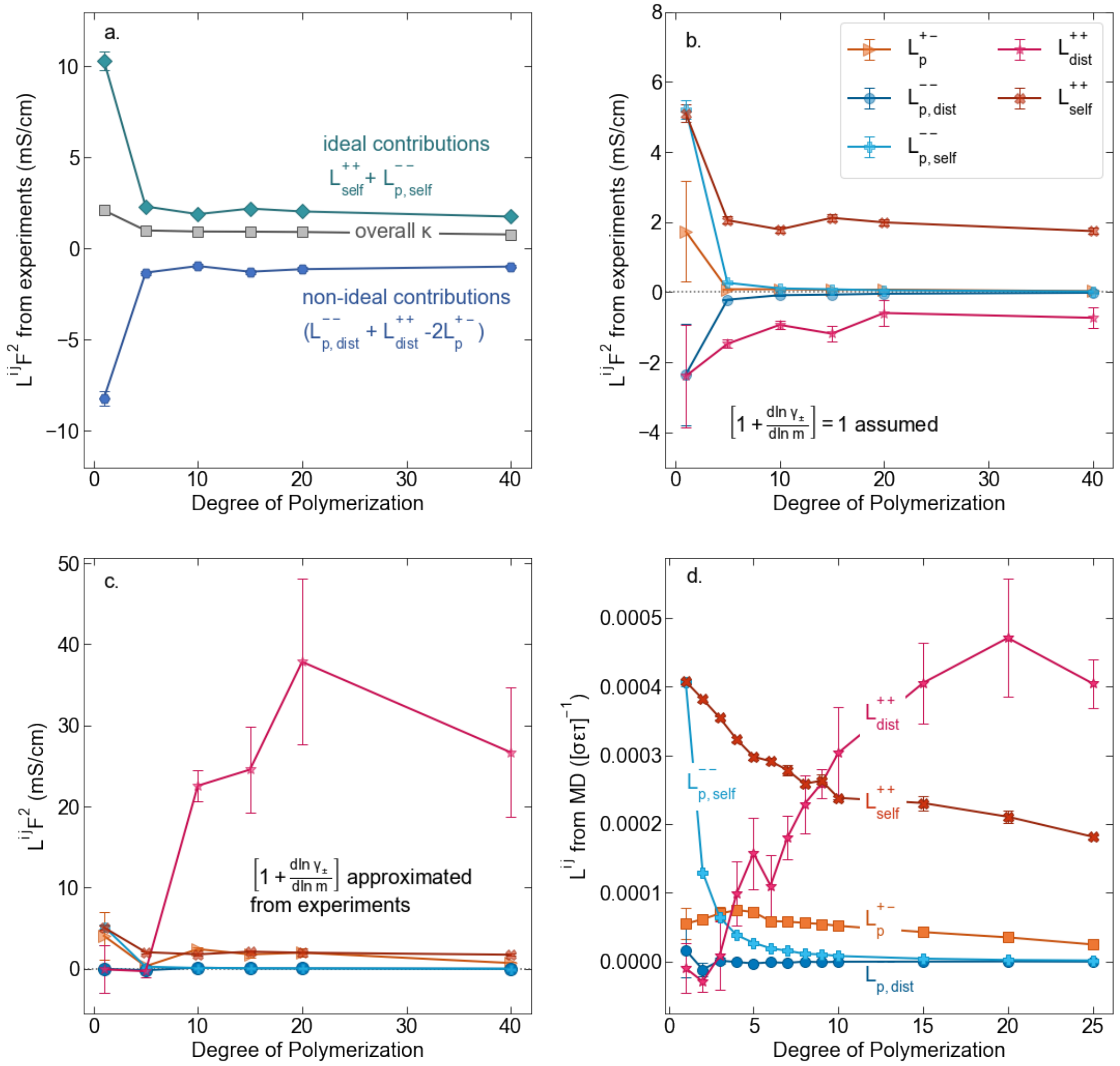

Figure S5: a) Onsager Transport Coefficients broken into ideal $[L^{++}_{\rm self} + L^{--}_{\rm p,self}]$ and non-ideal $[L^{++}_{\rm dist} + L^{--}_{\rm p,dist} - 2L^{+-}_{\rm p}]$ contributions to conductivity as calculated from experimental data (conductivity probe, eNMR, and restricted diffusion measurements) with no assumptions. Here the subscript p indicates the Onsager coefficients were calculated such that the anion is taken to be the entire polymer ($z_- = -N$) as opposed to an individual repeat unit ($z_- = -1$) used in the main text. b) Onsager Transport Coefficients calculated from experimental data assuming that $\left[\frac{\partial \ln \gamma_\pm}{\partial \ln m} + 1\right] = 1$. c) Onsager Transport Coefficients calculated using U-cell data for the thermodynamic factor. Note an approximate thermodynamic factor of 0.035 is used for polyanions with 10 or more repeat units, with thermodynamic factors for the monomer and 5 repeat unit polyanion given in Figure S4b. Legend is same as panel b. d) Onsager transport coefficients L^{ij} vs. degree of polymerization calculated with coarse grain molecular dynamics, reproduced from Ref. [11]. Note the molecular dynamics L^{ij} are all reported in Lennard Jones units corresponding to dimensions of (length * energy * time)$^{-1}$. Simulation data presented here corresponds roughly to a cation concentration of 0.48M.

References

[1] Christopher Barner-Kowollik. *Handbook of RAFT polymerization.* John Wiley & Sons, 2008.

[2] SR Simon Ting, Thomas P Davis, and Per B Zetterlund. Retardation in raft polymerization: Does cross-termination occur with short radicals only? *Macromolecules*, 44(11):4187–4193, 2011.

[3] Ming Chen, Graeme Moad, and Ezio Rizzardo. Thiocarbonylthio end group removal from raft-synthesized polymers by a radical-induced process. *Journal of Polymer Science Part A: Polymer Chemistry*, 47(23):6704–6714, 2009.

[4] Yen K Chong, Graeme Moad, Ezio Rizzardo, and San H Thang. Thiocarbonylthio end group removal from raft-synthesized polymers by radical-induced reduction. *Macromolecules*, 40(13):4446–4455, 2007.

[5] Christian H Hornung, Karin von Kanel, Ivan Martinez-Botella, Maria Espiritu, Xuan Nguyen, Almar Postma, Simon Saubern, John Chiefari, and San H Thang. Continuous flow aminolysis of raft polymers using multistep processing and inline analysis. *Macromolecules*, 47(23):8203–8213, 2014.

[6] Brooks A Abel and Charles L McCormick. "one-pot" aminolysis/thiol–maleimide end-group functionalization of raft polymers: Identifying and preventing michael addition side reactions. *Macromolecules*, 49(17):6193–6202, 2016.

[7] Javier Readáde Alaniz et al. Dual-pathway chain-end modification of raft polymers using visible light and metal-free conditions. *Chemical Communications*, 53(11):1888–1891, 2017.

[8] Fredrik Hallberg, István Furó, Pavel V Yushmanov, and Peter Stilbs. Sensitive and robust electrophoretic nmr: Instrumentation and experiments. *Journal of Magnetic Resonance*, 192(1):69–77, 2008.

[9] Qiuhong He and Zhaohui Wei. Convection compensated electrophoretic nmr. *Journal of magnetic resonance*, 150(2):126–131, 2001.

[10] Erik Pettersson, Istvan Furo, and Peter Stilbs. On experimental aspects of electrophoretic nmr. *Concepts in Magnetic Resonance Part A: An Educational Journal*, 22(2):61–68, 2004.

[11] Kara D Fong, Julian Self, Bryan D McCloskey, and Kristin A Persson. Onsager transport coefficients and transference numbers in polyelectrolyte solutions and polymerized ionic liquids. *Macromolecules*, 53(21):9503–9512, 2020.

[12] Kara D Fong, Helen K Bergstrom, Bryan D McCloskey, and Kranthi K Mandadapu. Transport phenomena in electrolyte solutions: Nonequilibrium thermodynamics and statistical mechanics. *AIChE Journal*, 66(12):e17091, 2020.

[13] Harnoor Singh Sachar, Nico Marioni, Everett S Zofchak, and Venkat Ganesan. Impact of ionic correlations on selective salt transport in ligand-functionalized polymer membranes. *Macromolecules*, 2023.

[14] John Newman and Karen E. Thomas-Alyea. *Electrochemical Systems.* John Wiley & Sons, Inc., 3 edition, 2004.

[15] Helen K Bergstrom, Kara D Fong, and Bryan D McCloskey. Interfacial effects on transport coefficient measurements in li-ion battery electrolytes. *Journal of The Electrochemical Society*, 168(6):060543, 2021.
